# Enhancement of Fixed-bed Flow Reactions under Microwave Irradiation by Local Heating at the Vicinal Contact Points of Catalyst Particles

**DOI:** 10.1038/s41598-018-35988-y

**Published:** 2019-01-18

**Authors:** Naoto Haneishi, Shuntaro Tsubaki, Eriko Abe, Masato M. Maitani, Ei-ichi Suzuki, Satoshi Fujii, Jun Fukushima, Hirotsugu Takizawa, Yuji Wada

**Affiliations:** 1 Department of Chemical Science and Engineering, School of Materials and Chemical Technology, Tokyo Institute of Technology, 2-12-1 E4-3 Ookayama, Meguro-ku, Tokyo 152-8552 Japan; 2 Research Center for Advanced Science and Technology, The University of Tokyo, 4-6-1, Komaba, Meguro-ku, Tokyo 153-8904 Japan; 3 Department of Information and Communication Systems Engineering, Okinawa National College of Technology, 905 Henoko, Nago-shi, Okinawa 905-2192 Japan; 4 Department of Applied Chemistry, Graduate School of Engineering, Tohoku University, 6-6-07 Aoba Aramaki, Sendai, Miyagi 980-8579 Japan

## Abstract

The formation of local high temperature regions, or so-called “hot spots”, in heterogeneous reaction systems has been suggested as a critical factor in the enhancement of chemical reactions using microwave heating. In this paper, we report the generation of local high temperature regions between catalyst particles under microwave heating. First, we demonstrated that reaction rate of the dehydrogenation of 2-propanol over a magnetite catalyst was enhanced 17- (250 °C) to 38- (200 °C) fold when heated with microwave irradiation rather than an electrical furnace. Subsequently, the existence of microwave-generated specific local heating was demonstrated using a coupled simulation of the electromagnetic fields and heat transfer as well as *in situ* emission spectroscopy. Specific high-temperature regions were generated at the vicinal contact points of the catalyst particles due to the concentrated microwave electric field. We also directly observed local high temperature regions at the contact points of the particles during microwave heating of a model silicon carbide spherical material using *in situ* emission spectroscopy. We conclude that the generation of local heating at the contact points between the catalyst particles is a key factor for enhancing fixed-bed flow reactions under microwave irradiation.

## Introduction

Microwaves (MW) have great potential for use in innovative chemical reaction processes, through enabling unique reaction fields that cannot be formed by conventional heating (CH). Their use in various chemical reactions including organic syntheses^1,2^, inorganic syntheses^3,4^, and heterogeneous catalytic reactions^5–7^ has been investigated since Gedye *et al*. reported the acceleration of chemical reactions under MW irradiation^8^. Chemical reactions promoted by MWs have been reported for both homogeneous and heterogeneous reaction systems. Various mechanisms for the enhancement of chemical reactions by MWs have been proposed, including new catalytic pathways^9^_,_ enhancement of dipole moments^10^, acceleration of electron transfer^11^, non-equilibrium local heating^12–14^, and others^15–18^.

MW heating has been also observed to accelerate fixed-bed flow reactions. Inhomogeneous distribution of temperature in the packed bed has been considered to be one of the key factors for reaction enhancement^19^. Accuracy of temperature measurement of the surface and inside the catalyst bed has been claimed to be critical for solid materials^20^. A fibre-optic thermometer measures temperature of the limited area inside the catalyst bed, while an infrared (IR) thermometer only measures the surface of the materials. Recently, generation of hot spots has been observed on the monolithic reactors by using an IR imaging and IR-transparent window to observe the temperature distribution at the surface of the irradiated objects^21,22^. In addition to inhomogeneous temperature gradients inside the catalyst bed, MWs are expected to affect thermodynamics or to induce micro-plasma to enhance rate of catalytic reactions. Zhang *et al*. applied MWs to an exothermic reaction (hydrodesulfurisation of thiophene) and an endothermic reaction (decomposition of hydrogen sulfide) over a MoS_2_/Al_2_O_3_ catalyst; the apparent equilibrium constants shifted favourably in the case of the endothermic reaction and unfavourably for the exothermic reaction due to the formation of hot spots in the catalyst bed^23^. They concluded that hot spot generation at the catalyst surface was responsible for the acceleration of the catalytic reactions^24^. Fidalgo *et al*. observed an improvement in the dry reforming of methane using MWs due to the formation of micro-plasma^25^. Stiegman *et al*. showed that MW irradiation promoted steam–carbon and related reactions by altering the thermodynamic parameters of these reactions^26^. The temperature distribution inside the catalyst bed is, however, should be carefully validated to evaluate the specific effects due to MW irradiation.

Simulation methods are useful for visualising the electromagnetic field and temperature distribution of a solid packed bed under MW irradiation. We previously demonstrated that a significant temperature gradient is generated in the packed bed under MW irradiation by combining experimental temperature measurements and the simulating the electromagnetic field distribution and heat transfer using a model catalyst bed represented by a uniform cylinder^27^. However, the temperature gradient alone could not fully explain the reaction enhancement; thus, we assumed that localised heating occurred in small regions of the packed bed. When a packed bed of particles is irradiated with MWs, each particle distorts the electromagnetic MW field^28^. Therefore, the electric field is concentrated where dielectric particles are close to each other. Previously, focused microwave electric fields have been used to sinter metal and ceramic powders^29,30^. Such highly localised electromagnetic fields may generate small non-equilibrium high temperatures regions and contribute to the enhancement of chemical reactions.

In this work, we first performed catalytic dehydrogenation of 2-propanol over a magnetite catalyst to experimentally study the effect of local high-temperature regions in the fixed-bed reactions. This endothermic reaction proceeds at relatively low temperature, and thus was a suitable model reaction for studying local heating under MW irradiation. Magnetite acted as both the MW susceptor and the dehydrogenation catalyst. We also used a single mode (TM_110_-mode) microwave resonator equipped with a semiconductor microwave generator to operate microwave irradiation with precisely controlled microwave frequency and power on the catalyst bed, because a wide and unstable oscillation spectrum of magnetron can cause fluctuation of electric field at the irradiated materials especially when equipped with a multi-mode cavity^31^. Subsequently, we carried out coupled simulations of the electromagnetic field distribution and heat transfer using a model of the packed beds to analyse the distribution of the electromagnetic field and the temperature in the beds under MWs. We also demonstrated the existence of high-temperature regions at the vicinal contact points of the spherical silicon carbide particles by *in situ* temperature measurements using a visible camera and an emission spectrometer.

## Results and Discussion

### Dehydrogenation of 2-propanol over magnetite catalysts

**Figure 1:**
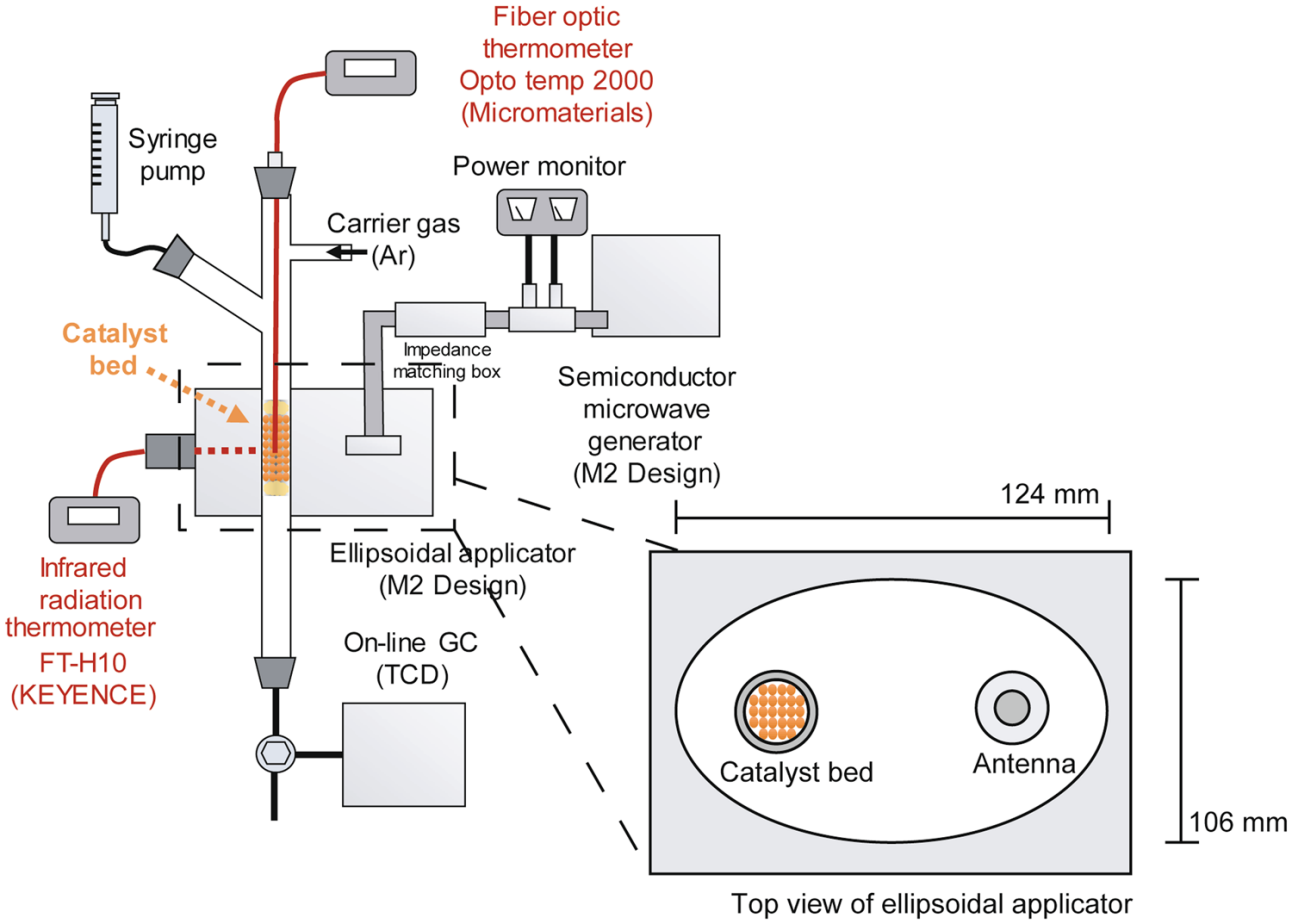
Schematic diagram of the 2-propanol dehydrogenation reaction.

The dehydrogenation of 2-propanol over the magnetite catalyst was carried out on the solid particles in the packed bed under MW irradiation using a single-mode elliptical applicator (TM_110_) equipped with a semiconductor MW generator at 2.45 GHz. (Fig. 1). The temperature of the side surfaces and core of the catalyst bed were monitored using an infrared radiation thermometer and fibre optic thermometer, respectively.

Figure S1 shows the dependence of the acetone yield on the time on stream at 250 °C using either MW or conventional heating (CH) by an electrical furnace for contact times of (a) 2.5 and (b) 20 seconds. The acetone yields remained constant until 20 minutes; the reactions were in a steady state from the beginning for both contact times. The acetone yields under MW heating were 19 and 12 times higher than under CH for contact times of 2.5 and 20 seconds.

**Figure 2:**
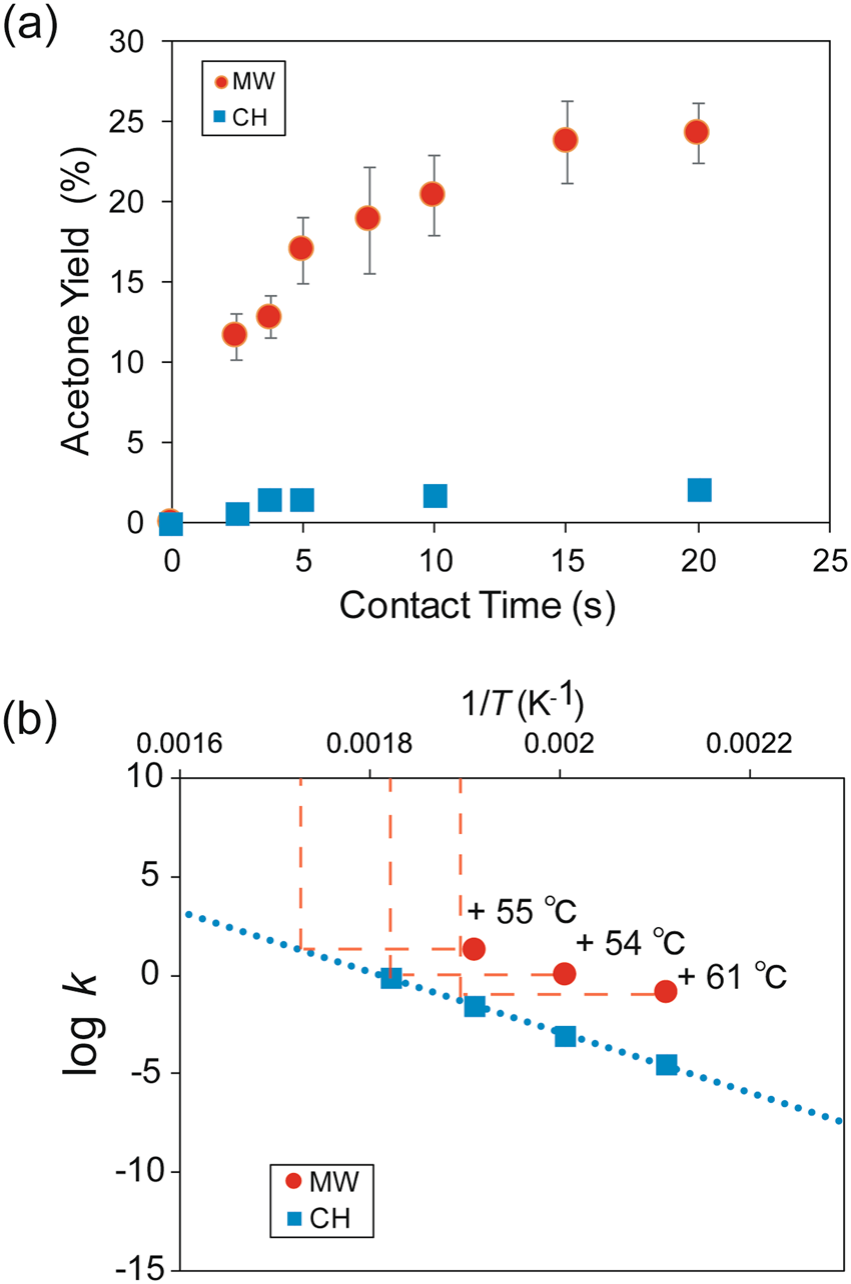
(**a**) The acetone yield as a function of the contact time under MW heating or CH at 250 °C. (**b**) Arrhenius plot of the dehydrogenation of 2-propanol over the magnetite catalyst under CH.

Figure 2a shows the yields of acetone as a function of contact time under MW heating or CH when the temperature of the side surface of the catalyst bed was 250 °C, as measured by an infrared radiation thermometer. The equilibrium conversion should reach almost 100% when the reaction proceeds at a temperature above 200 °C. In this reaction, however, the acetone yields did not reach the equilibrium conversion of 100% at 200–250 °C; the reaction was dominated by kinetics. Thus, the acetone yields increased with increasing contact time. The slope of yield versus contact time corresponds to the reaction rate constant in a differential reactor. The reaction rate constants under MW heating were 38, 22, and 17 times higher than those under CH when the temperature of the side surface of the catalyst bed was 200, 225, and 250 °C, respectively (Fig. S2). The activation energy and the frequency factor for CH were determined to be 127 kJ mol^−1^ and 1.10 × 10^10^ s^−1^, respectively (Fig. 2b). By applying these values to the reaction rates obtained under MW, the reactions under MW at 200 °C, 225 °C, and 250 °C were determined to correspond to CH temperatures of 255 °C, 279 °C, and 311 °C, respectively. We also detected a greater amount of carbon deposition during MW heating (Figs S2 and S3). From these results, we concluded that high-temperature regions were generated in the catalyst packed bed under MW, facilitating carbon deposition.

### Coupled Simulation of the Electromagnetic Field Distribution and Heat Transfer Using the FEM for the Sphere-Filled Catalyst Bed

The temperature distribution generated under MW heating in the solid-catalyst filled packed bed must be known to understand the rate enhancement effect of the MWs. Coupled simulations of the electromagnetic field distribution and heat transfer were conducted using the finite element method (FEM) in the COMSOL Multiphysics software (version 5.3, COMSOL Inc.) to determine the temperature distribution in the solid catalyst packed bed.

**Figure 3:**
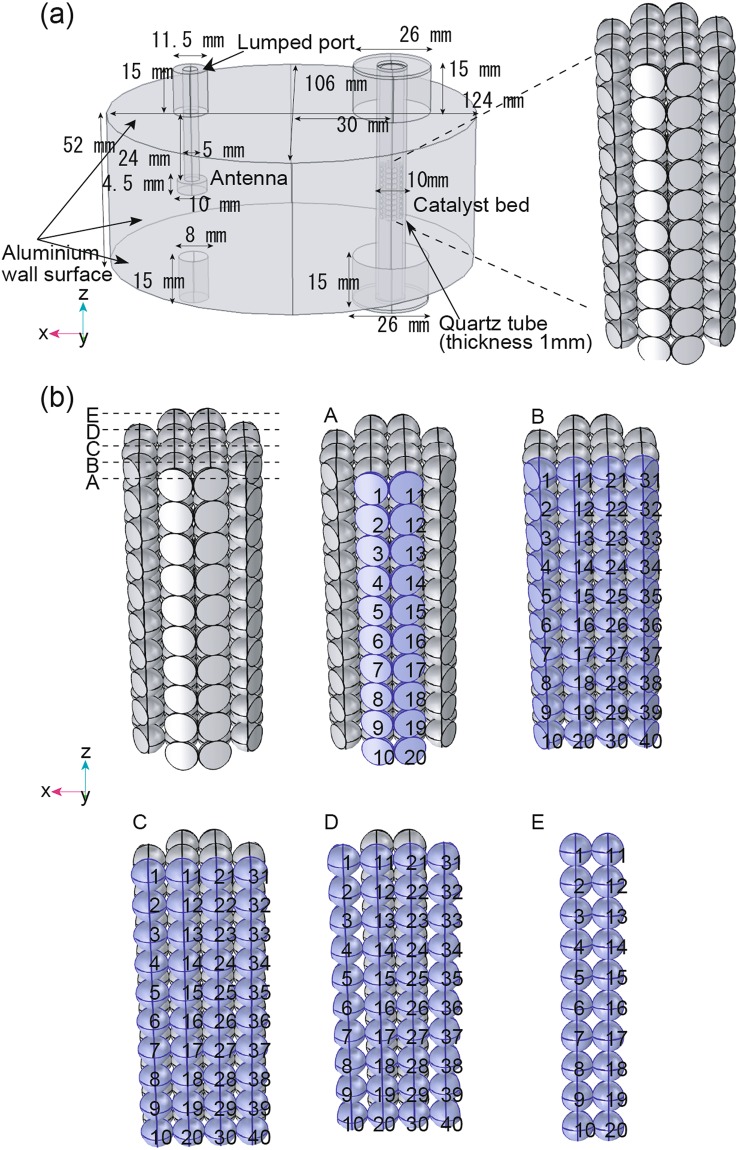
(**a**)The simulation model representing the inside of the elliptical applicator, including the antenna, quartz-tube, and model catalyst bed. (**b**) Sphere arrangement in the model catalyst bed. Spheres are classified and numbered along the XZ plane into the A-E planes. The A, B, C, D, and E planes contain 20, 40, 40, 40, and 20 spheres respectively. Spheres A1-20, B1-10, B31-40, D1-10, D31-40, and E1-20 were cut along the quartz tube to avoid overlap.

An orthogonal coordinate system was used in the simulation module. A model with the same dimensions as the experimental apparatus was reproduced in the simulation software (Fig. 3a). The model consisted of 160 spheres with a diameter of 1.9 mm arranged in a simple cubic lattice 19 mm in height and 8 mm in diameter (Fig. 3b). The calculation was conducted using a sphere diameter of 1.9 mm to reduce the computational load.

To investigate the effect of the vibration direction of the electric field on these distributions, the spheres were arranged so that their contact direction was completely perpendicular or parallel to the vibration direction. As in the actual experiments, the antenna and the model catalyst bed in the quartz tube were placed at the focal points of the elliptical applicator. The base of the antenna was connected to a lumped port that introduced the electromagnetic waves in the simulation module. The parameters necessary for the simulation are listed in Table S1. The MW output was set to 5 W, and the energy was transmitted to the applicator without loss by selecting the optimum characteristic impedance.

**Figure 4:**
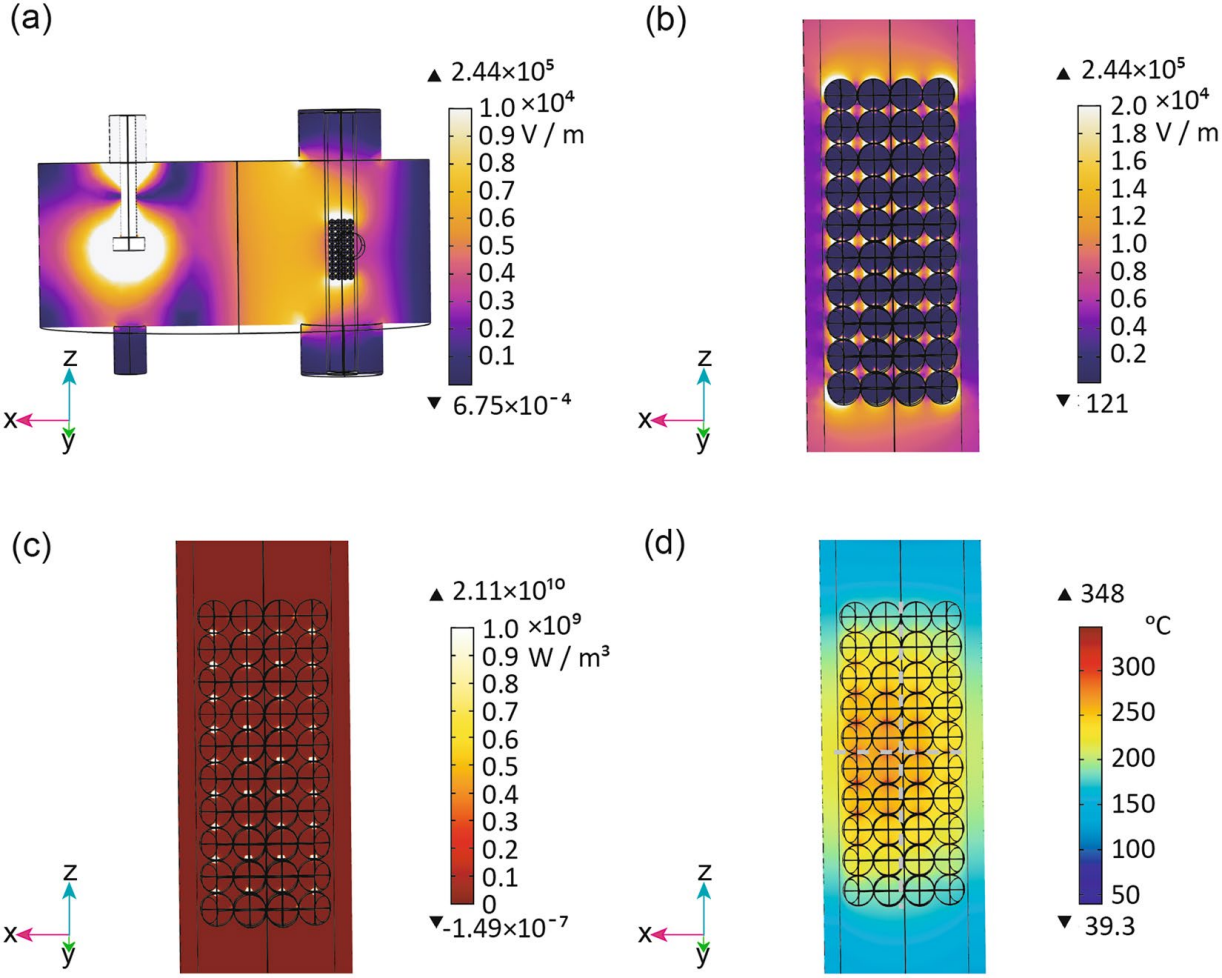
(**a**) Simulated electric field distributions in a cross-section of the microwave cavity including the C1-40 magnetite catalyst spheres, quartz tube, and antenna. Cross-sections of the simulated catalyst bed including the C1-40 magnetite catalyst spheres, void, and quartz tube showing the (**b**) electric field, (**c**) electromagnetic power loss density, and (**d**) temperature distributions. Input power: 5W.

Figure 4a shows the electric field distribution within the applicator. The electric fields were concentrated at the focal points of the ellipse by the shape of the MW chamber, intensifying the electric fields at the antenna and the upper and lower sides of the model bed. Around the model catalyst bed, the electric field near the antenna was stronger than that on the opposite side. A TM_110_ mode electromagnetic field was generated in the applicator, and the vibration direction of the electric field was the Z-axis.

In the enlarged view of Fig. 4b, the electric field inside of the spheres, at the contact point of the spheres, and in the voids near the contact points was approximately 500 V m^−1^, 35000 V m^−1^, and 230000 V m^−1^, respectively. The electric field strength in the magnetite spheres in the model catalyst bed was weakened by their high dielectric properties. The electric fields were concentrated at the contact points of the spheres in the Z-axis direction. The electric fields in the voids near the contact points of the spheres were intensified most (Fig. 4b). Therefore, an extremely high loss of electromagnetic power density occurred at the contact points (Fig. 4c). Figure 4d shows the temperature distribution in the model catalyst bed under MW irradiation. The temperature was high at the centre of the catalyst bed and decreased towards the edges. This temperature distribution resulted from heat dissipation at the edges of the catalyst bed.

**Figure 5:**
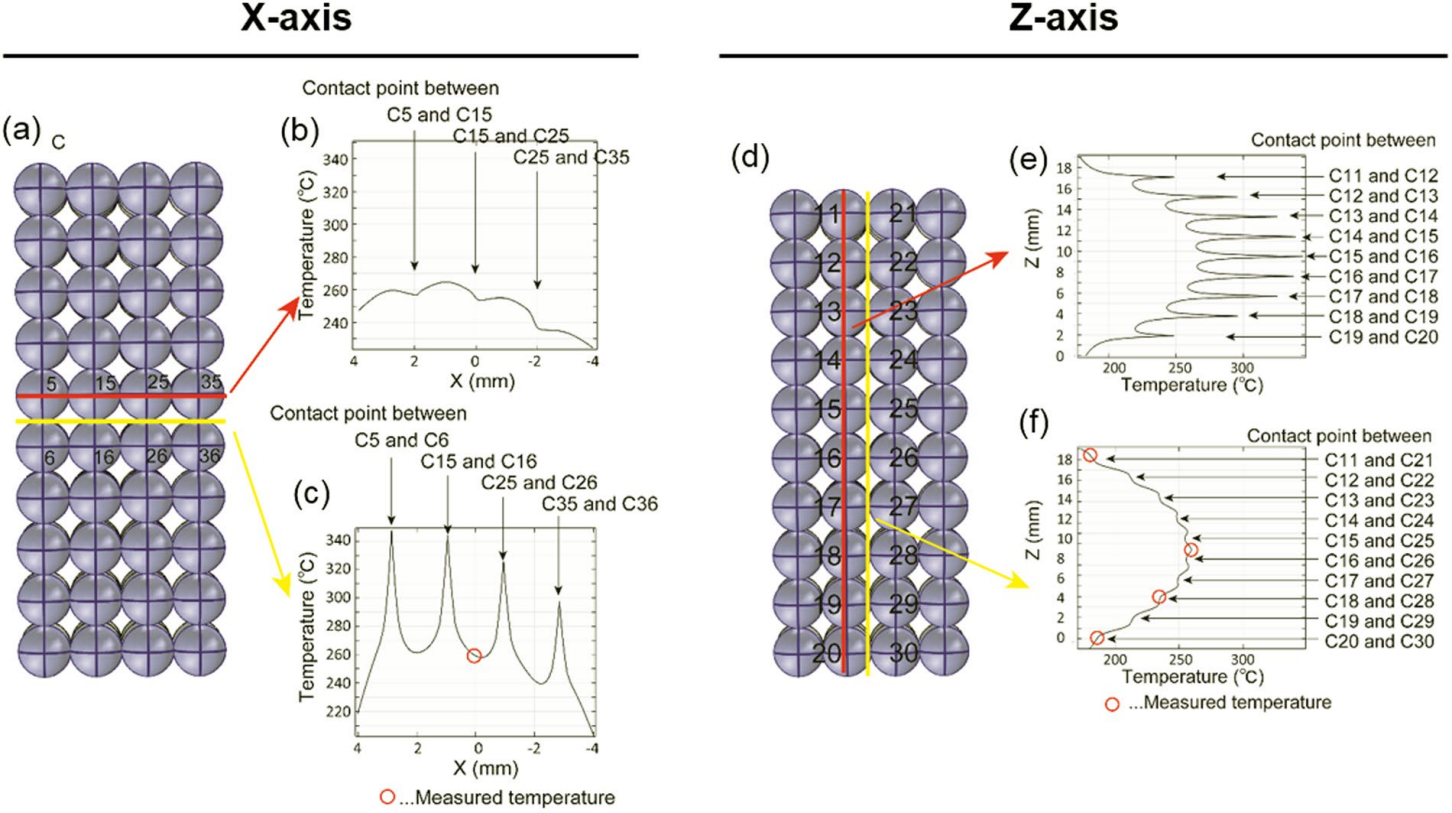
(**a**) Simulated temperatures along the X-axis of the model catalyst bed. Temperature distributions along the (**b**) red and (**c**) yellow lines in (**a**). (**d**) Simulated temperatures along the Z-axis of the model catalyst bed. Temperature distributions along the (**e**) red line and (**f**) yellow line in (**d**).

Figure 5a–c show the simulated temperatures along the X-axis of the model catalyst bed. The temperature distribution along the red line running through the spheres (Fig. 5b) formed a parabola with three dents. This parabola had an asymmetric, distorted shape with a maximum closer to the antenna side, because the electric field distribution was biased toward the antenna side. The locations of the three dents corresponded to the contact points of the spheres. As a result of heat dissipation from the surfaces of the spheres, the temperature at the centre of each sphere was higher than at its surface. Therefore, the temperature dropped at the contact points. Figure 5c depicts the temperature at the yellow line along the void between two rows of spheres (Fig. 5a). This temperature distribution had a broad parabolic shape with four evenly spaced spikes (Fig. 5c). The parabola had an asymmetric, distorted shape with the maximum closer to the antenna side. In addition, the locations of the high temperature spikes (297–344 °C) corresponded perfectly to the four contact points between the two rows of spheres

Figure 5d–f show the simulated temperatures along the Z-axis of the model catalyst bed. The temperature distribution along the red line through ten spheres and their contact points showed parabolic shape with nine evenly spaced spikes (Fig. 5e). The maximum temperature of the parabolic part of the curve occurred at the centre of the model catalyst bed. The locations of the temperature spikes corresponded perfectly to the contact points of the spheres. The temperature distribution along the yellow line through the void between two columns of spheres formed a parabola with ten dents (Fig. 5f). The temperature of parabolic curve reached a maximum at the centre of the model catalyst bed. The locations of the ten dents corresponded to the contact points of spheres.

To confirm the results of the temperature distribution simulation, the actual temperatures of the catalyst bed were measured at four points using a fibre optic thermometer at a side-surface temperature of 225 °C. The red circles in Fig. 5c,f indicate the measured temperatures. The experimentally measured temperatures were almost the same as the simulated temperatures.

**Figure 6:**
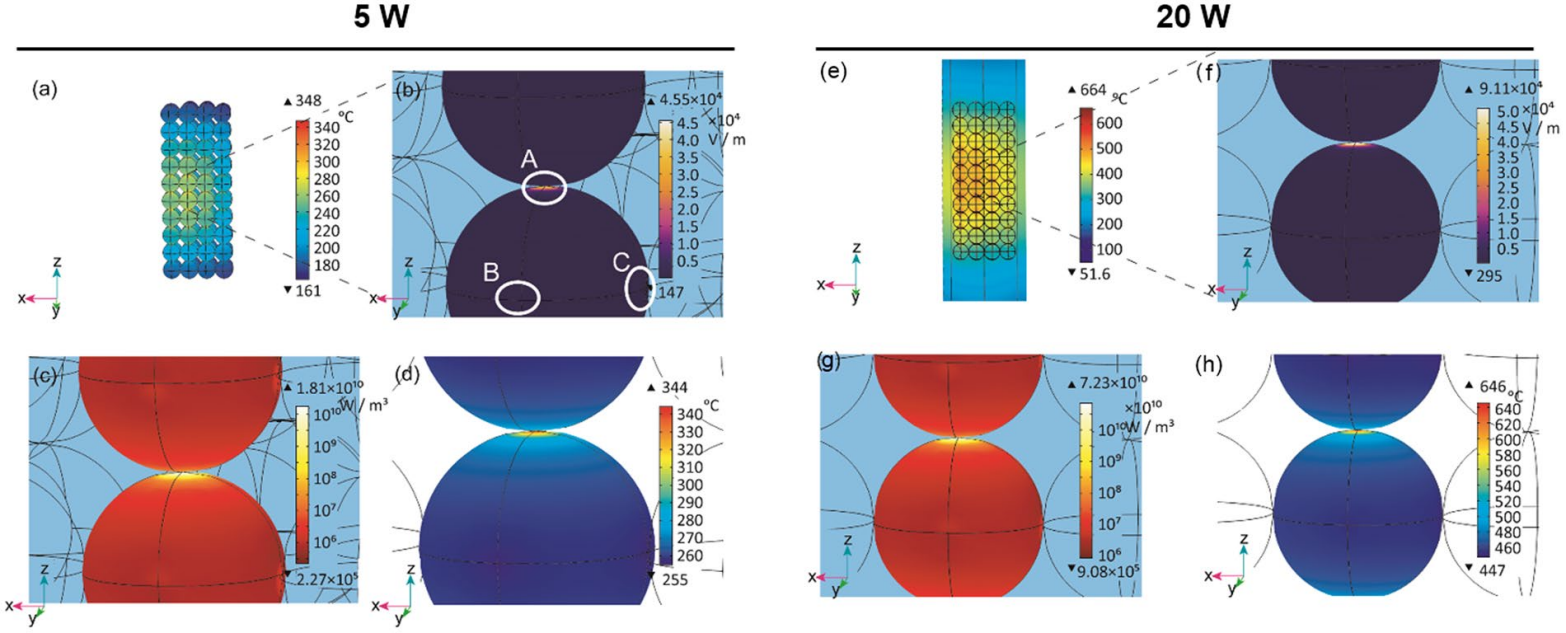
(**a**) The temperature distribution at the surface of the spheres in the C plane of the model catalyst bed. Simulated (**b**) electric field, (**c**) electromagnetic power loss density, and (**d**) temperature distributions at the surface of the magnetite catalyst spheres C15 and C16. The white circles in (**b**) labelled A, B, C indicate contact points between the spheres C15 and C16, C16 and B16, and C16 and C26, respectively. (**a**–**d**) Input power: 5 W. (**e**) The temperature distribution of a cross-section of the model catalyst bed. Simulated (**f**) electric field, (**g**) electromagnetic power loss density, and (**h**) temperature distributions at the surface of the magnetite catalyst spheres C15 and C16. (**e**–**h**) Input power: 20 W.

Figure 6a shows the surface temperatures of the spheres C1-40 during the reaction. The temperatures of C15 and C16 were the highest among all the spheres in the model catalyst bed. Figure 6b–d show the electric field, the electromagnetic power loss density, and the temperature distributions in the spheres C15 and C16, respectively. The electric field was less than 500 V m^−1^ at the surface of the spheres, except for at the contact points, the electric field at the contact point between C15 and C16 was 45500 V m^−1^ (Fig. 6b). The field was especially concentrated at the contact points of the spheres in the Z-axis direction. However, this concentration of the electric field was not observed at the contact points between the spheres C16 and C26, or C16 and B16 along the X and Y-axes (Fig. 6b). This was due to the TM_110_ mode formed by the applicator, in which the electric field oscillates along the Z-axis direction. The electric field concentration occurred when the oscillation of the electric field and the contact of the spheres were aligned in the same direction.

Due to the concentration of the electric field, remarkable heat generation by the MWs (electromagnetic power loss density) also occurred at the contact points (Fig. 6c). The electromagnetic power loss density was approximately 106 W m^−3^ at the surface of the spheres, except for at the contact point, where 1010 W m^−3^ of energy was converted to heat. As a result of the electric field concentration, the temperature of the regions at the contact points was increased 90 °C during MW heating (Fig. 6d).

To further understand the heat generation phenomenon at the contact points of vicinal particles, we investigated the influence changing the MW input power and the size of the spheres. First, the MW input power was increased from 5 to 20 W under the same conditions. Figure 6e–h show the electric field distribution, electromagnetic power loss density, and temperature distribution the spheres C15 and C16 at a MW input power of 20 W. The distribution was similarly to that at 5 W, with the highest temperature inside the catalyst bed, and decreasing temperatures toward the outside. (Fig. 6e). The electric field at 20 W was less than 1000 V m^−1^ at the surface of the spheres, except for at the contact point; this value was approximately twice as great as the corresponding 500 V m^−1^ field at 5 W (Fig. 6f). The electric field at the contact point between C15 and C16 was 91000 V m^−1^, which was also twice as great as the corresponding electric field of 45500 V m^−1^ at 5 W. The concentration of the electric field also caused remarkable intensification of the electromagnetic power loss density at the contact points (Fig. 6g). The values of the electromagnetic power loss density over the surface of the spheres at 20 W were four times greater than those at 5 W when compared at the same locations. As a result of the electric field concentration, high-temperature regions occurred at the contact points during MW heating (Fig. 6h). The temperature over 86% of the surfaces of C15 and C16 was approximately 447–480 °C under 20 W MW irradiation, while the micro-regions at the contact points in the Z-axis direction were maintained at a steady state of 646 °C. As a result, the local temperature at the contact points was 190 °C higher than the other regions. Thus, increasing the MW input power increased the temperature difference at the contact point.

We also investigated the behaviour of the electric field and temperature distribution in spheres with different diameters (2.4 mm and 400 μm). In order to investigate the electric field and temperature distribution with 2.4 mm spheres, a new model catalyst packed bed was constructed by arranging 64 spheres (Fig. S5). For the 400 μm spheres, a model representing the whole model catalyst bed would require an unrealistic computational time; therefore, the simulation was conducted using 125 spheres (Fig. S6).

Figure S5c-d shows the electric field and the temperature distribution of the spheres G12 and G13 in the model catalyst bed composed of 2.4 mm spheres (Fig. S5b) irradiated with 5 W MWs. The electric field was less than 400 V m^−1^ at the surface of the spheres excluding the contact point, where it was 42200 V m^−1^ (Fig. S5). The local temperature at the contact points was 153 °C higher than the other regions  (Fig. S5d). The degree of concentration of the electric field was almost the same for the 1.9 and 2.4 mm spheres; however, the temperature increase at the contact point was larger for the 2.4 mm sphere. Local heating at the contact points was also observed in the simulation involving 400 μm spheres (Fig. S6). This diameter was similar to those of the particles used in the actual experiments. The MW heating generated hot spots were 80 °C hotter than the other regions  (Fig. S6c).

The increased temperature differences in the larger spheres were due to heat conduction. Thermal conductivity contributes significantly to the formation of the high-temperature regions at the particle contact points. The value of the thermal conductivity used in this simulation was lower than that of the sintered magnetite, because the value of compacted magnetite powder was used in the simulation. Therefore, a simulation was also executed using the thermal conductivity of sintered magnetite (4.23–1.37 × 10^−5^ × T [W m^−1^ C^−1^])^32^. Altering the thermal conductivity changed the temperature distribution in the spheres (Fig. S7). The temperature distribution over the entire model catalyst bed was similar to that in Fig. 3. However, the temperature at the contact points was only 15 °C higher than the other regions (Fig. S7b); the higher thermal conductivity led to a smaller temperature difference.

Figure S8 shows the results of a simulation using a face-centred cubic lattice instead of the simple cubic lattice. In this lattice, the contacts between the particles are not perfectly parallel to the Z-axis, along which the electric field vibrates. The face-centred cubic lattice has a coordination number of 12. Focusing on one sphere, no electric field concentration occurred at the four contact points that were completely perpendicular to the Z-axis, however, the electric field was concentrated at the eight contact points having a Z-axis component (Fig. S8c). As a result, eight high-temperature regions were observed for each sphere (Fig. S8d). Therefore, local high-temperature regions can be generated at the contact points having a component in the electric field vibration direction, even if they are not perfectly parallel.

### Direct Observation of the Temperature Distribution of the Silicon Carbide Spheres

**Figure 7:**
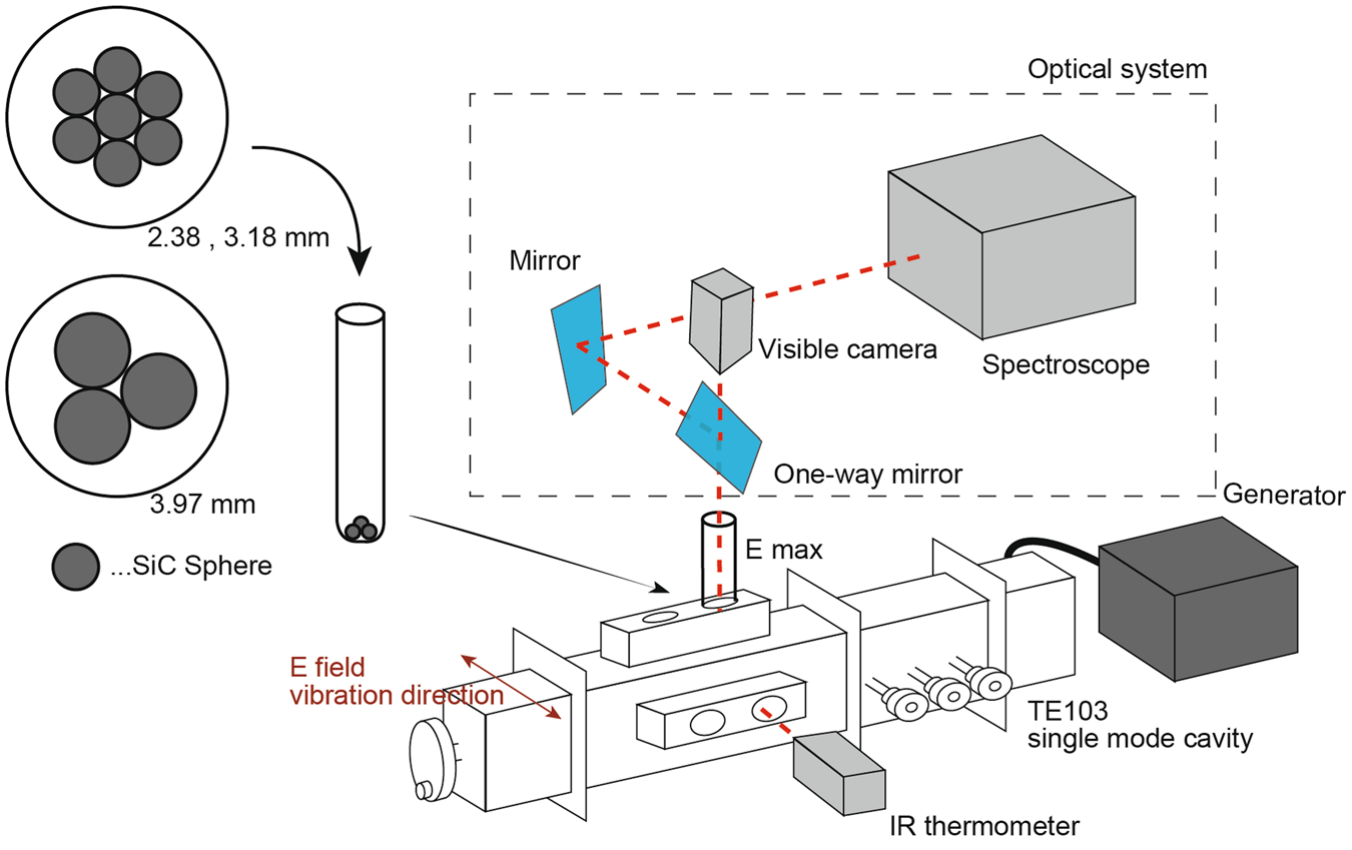
Setup for the MW heating experiments and the *in situ* emission spectroscopy of the SiC spheres.

An *in situ* MW emission spectroscope equipped with a visible camera was used to directly determine whether the increased temperature at the contact points predicted in the simulation could actually be observed under MW irradiation using the TE_103_ waveguide applicator (Fig. 7). By using perfect spheres, the influence of complicated shapes of the real catalyst particles such as edges is eliminated. SiC spheres were used for this experiment because perfectly spherical magnetite was not available. SiC is a suitable material for heating experiments because it can withstand high temperatures and is easily heated by MWs. The diameters of the SiC spheres were 2.38, 3.18 and 3.97 mm, which were similar to those used in the simulation.

We observed the light emission due to the thermal radiation from the MW-heated SiC spheres. A temperature of higher than 1000 °C was necessary to observe the light emission with the visible camera. When two SiC spheres were heated, the temperature did not reach 1000 °C even at a MW input power of 200 W. By increasing the number of SiC spheres, a higher temperature was obtained at the same input power. Seven 2.38 or 3.18 mm spheres were needed to reach 1000 °C, while three 3.97 mm spheres were sufficient.

**Figure 8:**
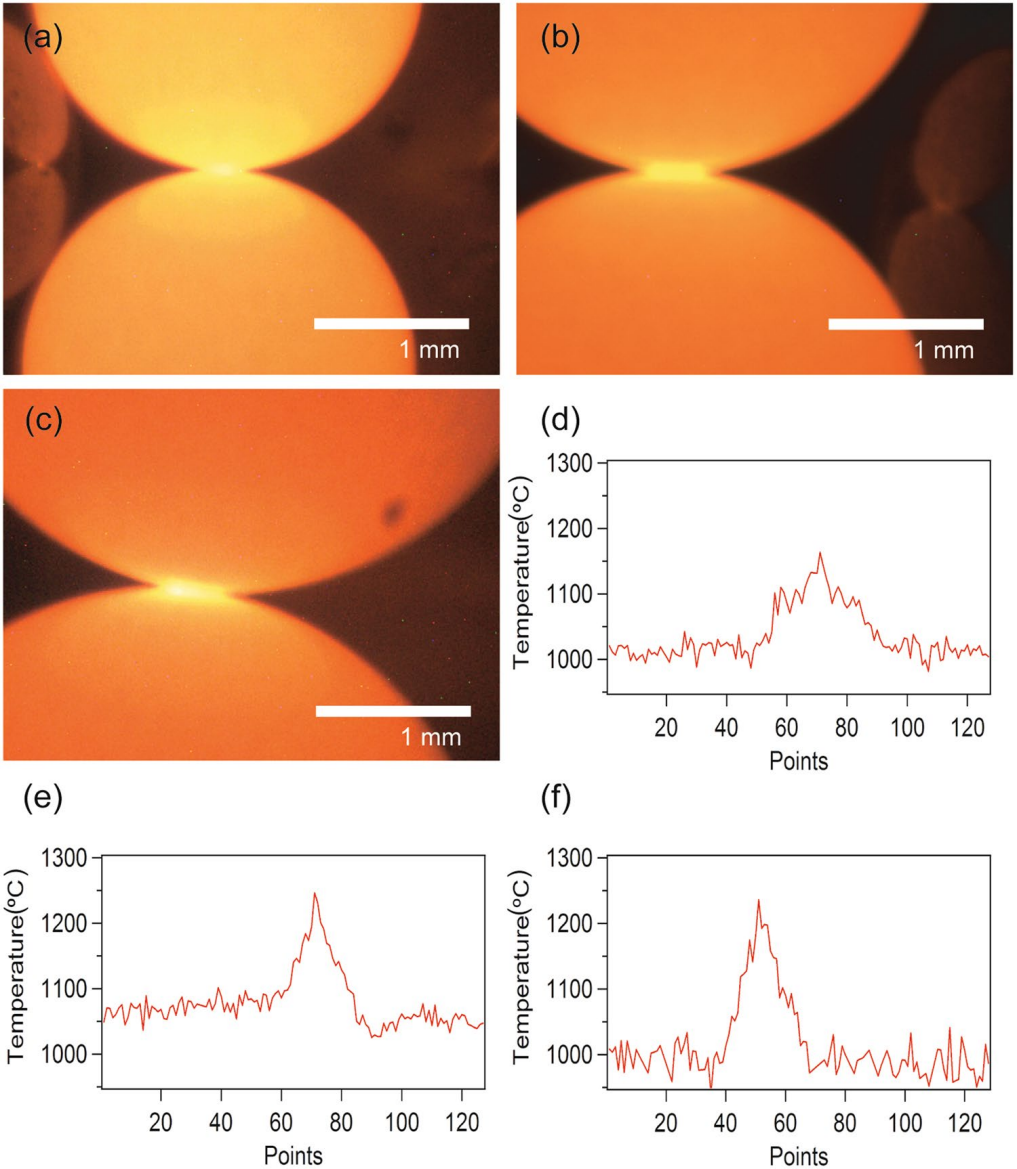
Visible light images of two SiC spheres in contact with each other. The diameters of the SiC spheres were (**a**) 2.38, (**b**) 3.18, and (**c**) 3.97 mm, respectively. The temperature distributions of the (**d**) 2.38, (**e**) 3.18, and (**f**) 3.97-mm diameter SiC spheres along the contact points of two samples, as determined by the light intensity. These locations are indicated by the dotted black lines in (**a**–**c**). The visible camera can observe a 3.2 mm × 2.4 mm region.

Figure 8a shows the visible camera image of the two most strongly heated 2.38 mm spheres under 85 W of MW power. The two SiC spheres emitted light via thermal radiation, with the brightest light being observed at the contact point of the spheres. The intensity of the brightness was dependent on the temperature; thus, a high temperature region occurred at the contact point between the SiC spheres. Figure 8d shows the calculated temperature distribution obtained from the intensity ratio of light with a wavelength of 650 to 800 nm measured by the emission spectrometer along the dotted line in the Fig. 8a. The temperature increased in a 0.5 mm region around the contact point. The maximum temperature at the contact point was 1150 °C (Fig. 8d), and the temperature difference was about 150 °C between the contact point and the other regions of the spheres.

Figure 8b shows the image from the visible camera of the two most strongly heated of the seven 3.18 mm spheres under 67 W of MW irradiation. The region at the contact point between the spheres was the brightest, similarly to the 2.38 mm spheres. The maximum temperature of 1250 °C was attained at the contact point (Fig. 8e). Over the rest of the surface of the spheres, the temperature was almost constant at 1050 °C, representing a difference of approximately 200 °C from the contact point.

Figure 8c and Movie S1 shows an image and video from the visible camera of the two most strongly heated 3.97 mm spheres under 98 W of MW irradiation. The brightest region was again observed at the contact point, and corresponded to the maximum temperature of 1240 °C (Fig. 8f). Over the rest of the surface of the spheres, the temperature was almost constant at 1000 °C, a difference of approximately 240 °C.

When the 2.38, 3.18, and 3.97 mm SiC spheres were heated by MWs, the temperature differences between the contact point and the other regions of the spheres were 150 °C, 200 °C, and 250 °C, respectively. Therefore, the difference in the temperature between the contact point and the rest of the sphere was strongly dependent on the size of the SiC sphere.

The *in situ* emission spectroscopy results demonstrated the generation of a local high temperature region at the vicinal contact point of particles during MW irradiation. The temperature difference between the contact point and the rest of the surface area reached approximately 240 °C. The local high temperature regions between the catalyst particles is therefore concluded to be a key factor in the enhancement of the fixed-bed flow reaction.

## Conclusions

We carried out the dehydrogenation of 2-propanol over a magnetite catalyst as a model reaction to investigate the effects of MW irradiation on the reaction rate enhancement. The reaction rate constants of the dehydrogenation of 2-propanol under MWs were 38, 22, and 17 times higher than those obtained using an electrical furnace at 200 °C, 225 °C, and 250 °C, respectively. The electric field and temperature distributions in the catalyst bed were analysed by a finite element method simulation using model catalyst beds consisting of catalyst spheres in an ordered arrangement. The electric fields were concentrated at the contact points of the spheres along the direction of the oscillating electric field. The concentrated electric fields led to local high temperature regions at the contact points of the spheres. We also demonstrated the existence of local high-temperature regions at the contact points of solid particles under MW irradiation using *in situ* emission spectroscopy. The brightest light from thermal radiation was observed at the contact point between the SiC spheres, demonstrating that local high temperature regions are indeed generated at the contact points of the particles. The present results clearly confirm that the local high temperature regions occurred at the vicinal contact points between the catalyst particles under MW irradiation. These local high temperature regions can be exploited to enhance fixed-bed flow reactions.

## Methods

### Materials

Magnetite (Kojundo Chemical Laboratory Co., Ltd.) was used as a catalyst for the dehydrogenation of 2-propanol. The magnetite powder was compacted at 10 MPa to 13 mm (diameter) × 4 mm (height) to obtain pellets. The magnetite pellets were crushed and sieved to obtain the magnetite catalysts (250–710 μm grains). The particle size distribution and SEM image of the magnetite catalyst are shown in Fig. S9. 2-Propanol was purchased from Wako Pure Chemical Industries, Ltd.

### Dehydrogenation of 2-Propanol over the Magnetite Catalyst under MW irradiation

Dehydrogenation of 2-propanol was performed using a magnetite catalyst (2.0 g) in a quartz reactor with an inner diameter of 8 mm (Fig. 1). 2-Propanol was supplied to the reactor using a feeder with argon as the carrier gas with a constant partial pressure at each flow rate (Table S2). 2-Propanol was gasified by preheating to 110 °C with a heater at the exit of the feeder. The magnetite catalyst was heated by MWs in an aluminium elliptical applicator equipped with a quarter-wavelength dipole antenna at one focal point of the ellipsoid (Chronix, Inc.). A TM_110_ single mode was formed in the elliptical applicator^33^ equipped with a semiconductor microwave generator (2.45 GHz). The quartz reactor was then placed at the other focal point. Electromagnetic wave energy was focused on the catalyst bed at the focal point of the ellipsoid to efficiently heat the catalysts. The temperature of the side surface of the catalyst bed was measured using an infrared radiation thermometer (FT-H10, KEYENCE Co.). The products in the gas phase were sampled every 5 min and analysed using an on-line gas chromatograph (GC-8A with a thermal conductivity detector, Shimadzu Co.) with a BX-10 column (length: 3 m, inner diameter: 3 mm, mesh range: 60/80, GL Science Inc.). The same reaction was carried out under the same conditions using an electric heating furnace (130 mm (diameter) × 170 mm (height)). The surface products at the magnetite catalyst were examined by CHNS analysis (vario MICRO cube, Elementar) and a Fourier transform infrared spectrometer (FTIR-6600, JASCO Co.) before and after the reactions (both MW and CH) to determine the quantity of coke deposition. The shape and particle size of the catalyst were observed with a scanning electron microscope (VE-8800, KEYENCE Co.).

### Parameters for Coupled Simulations of the Electromagnetic Field Distribution and Heat Transfer for a Sphere-Filled Catalyst Bed Using a Finite Element Method

The permittivity and permeability of the magnetite were measured by a perturbative method using a vector network analyser (ZND, 100 kHz to 8.5 GHz, ROHDE&SCHWARZ) and a TM_010_ mode resonator. According to the literature^34^, the permittivity and permeability are nearly the same at room temperature and 300 °C (7.8–0.39 j at room temperature, 8.1-0.50 j at 300 °C, and 2.7-1.6 j at room temperature, 2.5–1.8 j at 300 °C, respectively). Thus, the permittivity and the permeability measured at room temperature could be used in the simulation without greatly affecting the results.

The electrical conductivity of the magnetite was measured using a powder resistivity measurement system (MCP-OD51, MITSUBISHI CHEMICAL ANALYTECH) with a four-point probe. The electrical conductivity of magnetite scarcely changes even when heated to 300 °C^35^. The room-temperature electrical conductivity value at room temperature was used in the simulation. The density of the magnetite was obtained by dividing the weight of a magnetite pellet by its volume.

The heat capacity and thermal conductivity of the magnetite were obtained using differential scanning calorimetry (Q100 DSC, TA Instruments) and a laser-flash method using a laser flash apparatus (LFA 447, NETZSCH), respectively. The heat capacity and the thermal conductivity values of the magnetite catalyst were measured at 200 and 300 °C, respectively, and a function that linearly approximated these two values at different temperatures was used to set these parameters in the model catalyst bed particles in the simulation.

The parameter values of aluminium metal were applied as the parameters of the chamber wall in the simulation model, and the wall was set as the impedance boundary condition. The parameter values of quartz glass and argon were applied for the reaction tube and reaction gas in the simulation model, respectively. For the parameter values of aluminium, argon, and quartz glass, literature data from the database of COMSOL Multiphysics simulation were used. The initial temperature of the gas flow in the reaction tube was set to 110 °C in the simulation because the reaction gas was preheated to 110 °C for the actual dehydrogenation reaction.

### Direct Observation of Specific Local Heating at the Contact Points of SiC Spheres by Microwave *in situ* Emission Spectroscopy

*In situ* emission spectroscopy was used for the direct observation of local high temperature regions at the contact points of the particles. Perfectly spherical SiC was used as a model material to reflect the simulation models. SiC spheres with diameters of 2.38, 3.18, and 3.97 mm (Sato Tekko Co., LTD.) were used as model spherical catalyst particles. The permittivity and tan δ of SiC were 7.9 and 0.33 respectively, and the permeability and tan δ of SiC were 1.0 and 0 respectively. The permittivity and tan δ of magnetite were 9.8 and 0.11 respectively, and the permeability and tan δ of magnetite were 2.0 and 0.031, respectively. Therefore, both SiC and magnetite generate heat mainly by dielectric loss, so the phenomena seen in magnetite can also be observed in SiC.

The spheres were brought into contact with each other by placing seven spheres in the case of the 2.38 and 3.18 mm spheres and three spheres in the case of the 3.97 mm spheres in a quartz test tube with a round bottom (Fig. 7). Each sample was heated in the atmosphere at the electric field maximum point of the TE_103_ mode cavity. The heated spheres were monitored using a microscopic imaging system (MISM-08, Mutsumi Corporation)^36^. The temperature distributions of the spheres were calculated from the emission spectrum at the wavelengths from 629 to 814 nm. The visible camera can observe a 3.2 mm × 2.4 mm region in steps of 5 μm.

The impurities on the surfaces of each SiC sphere were analysed using an electron probe microanalyser (EPMA, JXA-8200, JEOL Ltd.) to confirm that there were no differences, other than the size, among the SiC spheres (Fig. S10). Although a slight amount Fe was present on the surface of the spheres with a diameter of 3.97 mm, no Fe was present in the cross-section of any of the spheres. Since the Fe content was small, its influence on heat generation was negligible.

## Electronic supplementary material

Supplementary information

Movie S1
