# Supplementary material for: Enhancement of Fixed-bed Flow Reactions under Microwave Irradiation by Local Heating at the Vicinal Contact Points of Catalyst Particles
Source: Sci Rep. 2019 Jan 18;9:222. doi: 10.1038/s41598-018-35988-y (PMC6338740; doi:10.1038/s41598-018-35988-y)
Supplement: Supplementary file 1 — Supplementary information [file 41598_2018_35988_MOESM1_ESM.pdf]

Supplementary information

Enhancement of Fixed-bed Flow Reactions under Microwave Irradiation by Local Heating at the Vicinal Contact Points of Catalyst Particles

Naoto Haneishi[1], Shuntaro Tsubaki[1], Eriko Abe[1], Masato M. Maitani[2], Eiichi Suzuki[1], Satoshi Fujii[1,3], Jun Fukushima[4], Hirotsugu Takizawa[4], & Yuji Wada[1]

1. Department of Chemical Science and Engineering, School of Materials and Chemical Technology, Tokyo Institute of Technology, 2-12-1 E4-3 Ookayama, Meguro-ku, Tokyo 152-8552, Japan
2. Research Center for Advanced Science and Technology, The University of Tokyo, 4-6-1, Komaba, Meguro-ku, Tokyo 153-8904, Japan
3. Department of Information and Communication Systems Engineering, Okinawa National College of Technology, 905 Henoko, Nago-shi, Okinawa 905-2192, Japan
4. Department of Applied Chemistry, Graduate School of Engineering, Tohoku University, 6-6-07 Aoba Aramaki, Sendai, Miyagi 980-8579, Japan

Table S1. Parameters used in the coupled simulation of the electromagnetic field distribution and heat transfer.

Materials	ε^*	μ^*	σ [S m^{-1}]	ρ [kg m$^{-3]}$	Cp [J g^{-1} °C^{-1}]	k [W m^{-1} °C^{-1}]
Catalyst bed	9.8 -1.1j	2.0 -0.062j	17.2	2840	0.00057 × T + 0.5354	0.0016 × T – 0.4168
Quartz glass	2.09	1	0	2203	0.703	1.38
Argon	1	1	0	522 / T	0.52	a + b × T + c × T2 + d × T3
Aluminum	1	1	3.774 × 107	-	-	-

ε* is the complex permittivity, μ* is the complex permeability, σ is the electric conductivity, ρ is the density, *C*p is the heat capacity, and *k* is the effective thermal conductivity. a: -2.47×10^{-4}, b: 7.37×10^{-5}, c: 5.23×10^{-8}, and d: 2.23×10^{-11}.

Table S2. Flow rates at each contact time.

Contact time (s)	2.5	3.8	5.0	7.5	10	20
2-Propanol ($mol\ h^{-1}$)	0.276	0.184	0.138	0.092	0.069	0.035
Argon ($mol\ h^{-1}$)	0.450	0.300	0.225	0.150	0.113	0.056

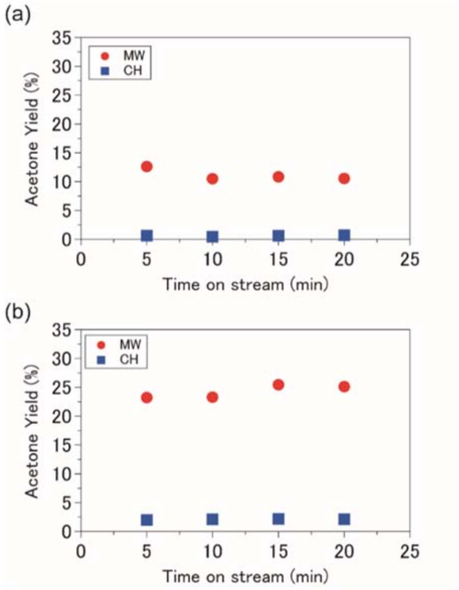

Fig. S1. The yields of acetone with respect to time on stream at 250 °C under MW heating or conventional heating (CH) for contact times of (a) 2.5 and (b) 20 seconds.

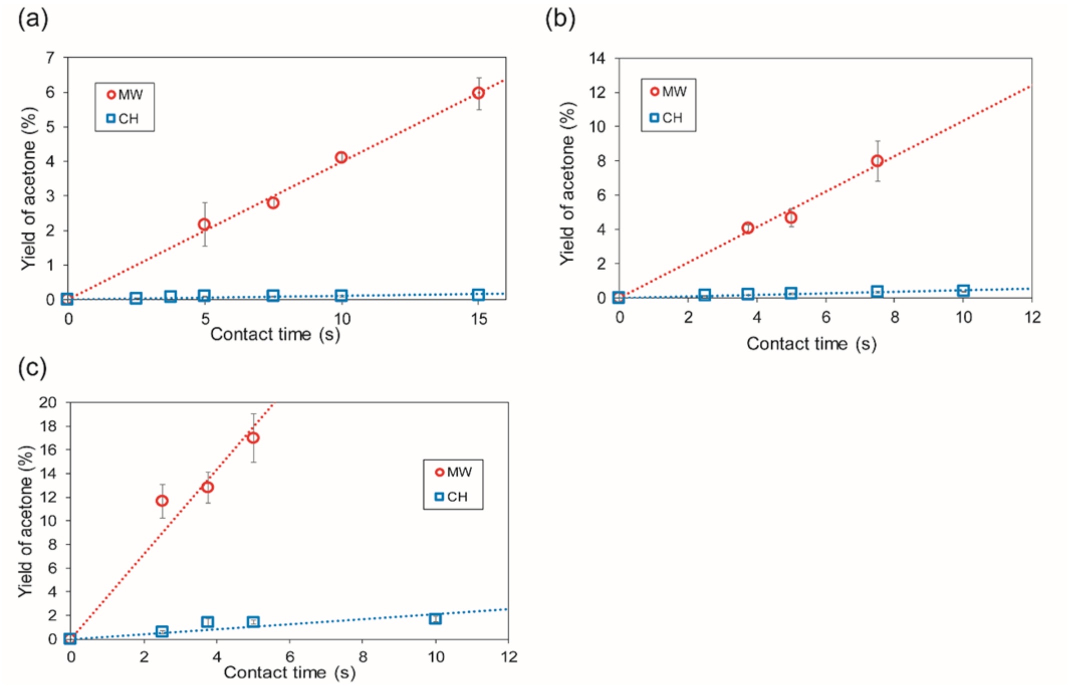

Fig. S2. The initial yields of acetone as a function of the contact time under MW heating or CH at reaction temperatures of (a) 200, (b) 225, and (c) 250 °C.

To satisfy the conditions of a differential reactor, the yield should be smaller than about 5%. However, the acetone yields at 250 °C under MW irradiation exceeded 10% at each contact time (Fig. S1). The contact time of the reaction under MW heating cannot be shortened because changing the amount of catalyst should be avoided. The amount of catalyst would affect the distribution of MWs.

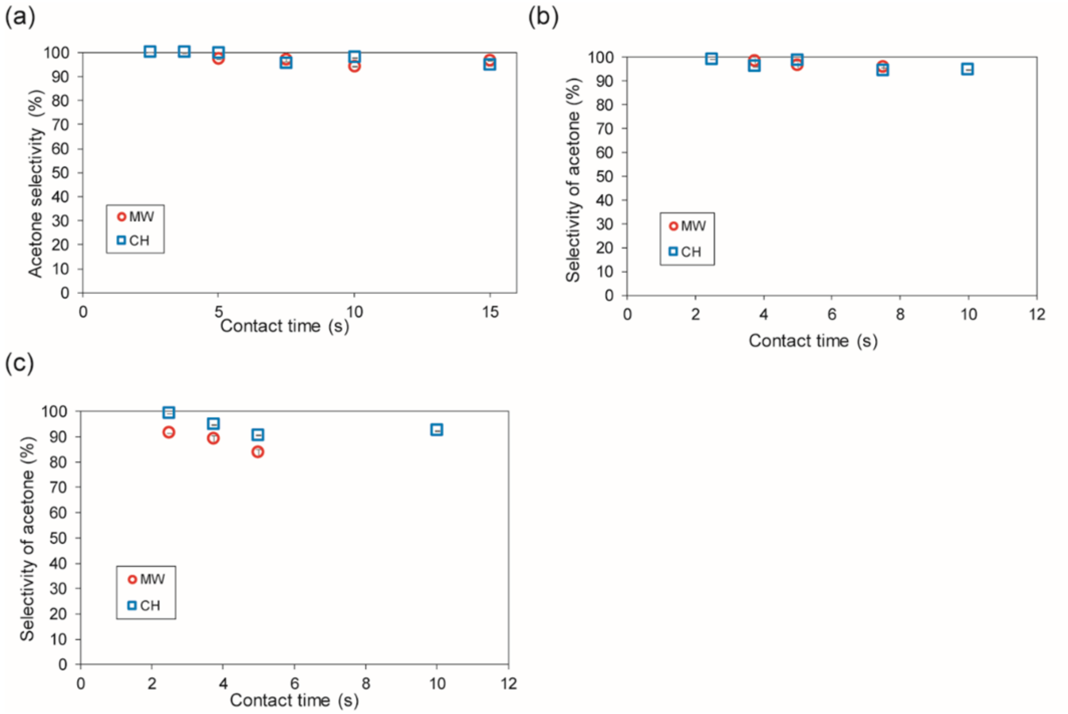

Fig. S3. The acetone selectivity as a function of contact time under MW heating or CH at reaction temperatures of (a) 200, (b) 225, and (c) 250 °C.

Although high acetone selectivity was obtained without generation of propylene (Fig. S2), carbon deposition occurred in the magnetite catalyst bed. By measuring the IR spectrum of the catalyst before and after the reaction at 250 °C, C–C bond was detected due to the carbon deposition (Fig. S3). In addition, when the amount of carbon deposition was quantified by CHNS analysis, the amount of carbon in the catalyst was found to have increased after MW heating to a greater extent than in the catalyst after CH at a reaction temperature of 250 °C (Fig. S3).

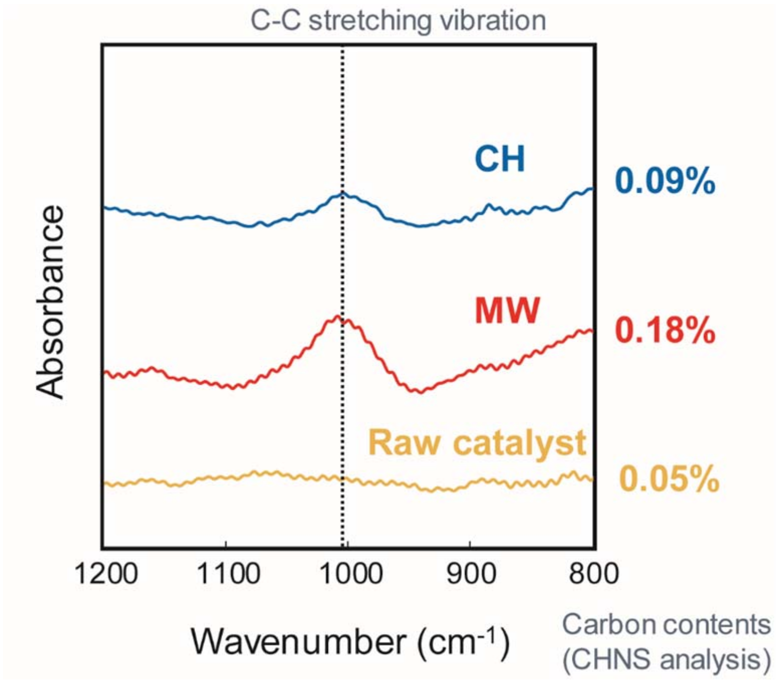

Fig. S4. FT-IR spectra and CHNS analysis of Fe_3O_4 catalyst before and after reaction at 250 °C.

The higher absorbance due to C-C stretching bond was observed for Fe_3O_4 after microwave reaction than conventional heating. CHNS analysis indicated that the amounts of carbon deposition in Fe_3O_4 were 0.18% for microwaves and 0.09% for conventional heating.

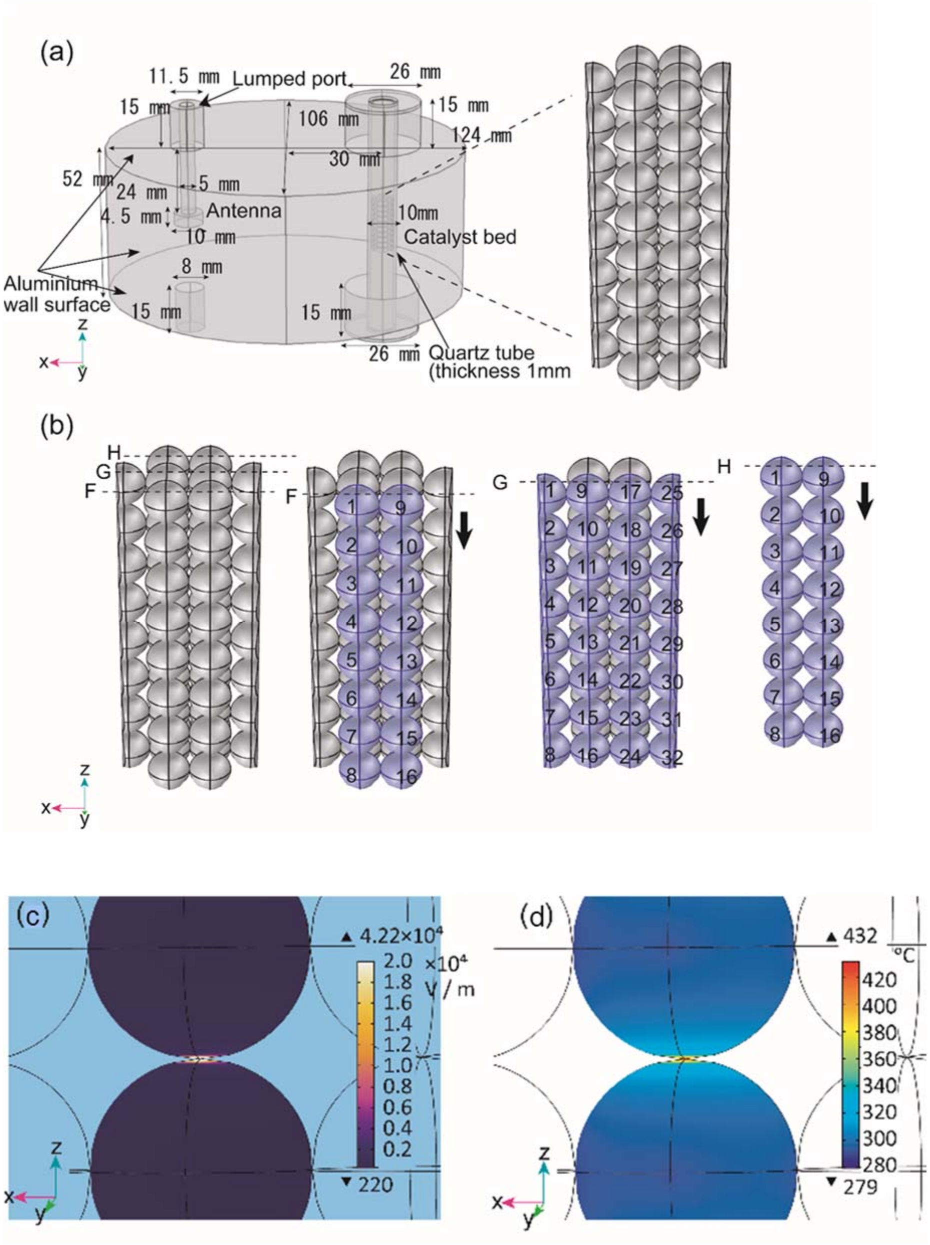

Fig. S5 (a) The simulation model duplicated the inside of the elliptical applicator including the antenna, the quartz-tube, and the model catalyst bed with diameter of 2.4 mm. (b) Detailed arrangement of spheres in the model catalyst bed. Each sphere is classified and numbered along the XZ plane into the F-H planes. The F, G, and H planes contain 16, 32, and 16 of spheres respectively. The spheres of G1-8, and G25-32 are cut along the shape of the quartz tube so that the spheres did not overlap the quartz tube. Simulated (c) electric field and (d) temperature distributions at the surface of two the magnetite catalyst spheres of G12 and G13 shown in Fig. S5b with a diameter of 2.4 mm in the model catalyst bed. The input power: was 5 W.

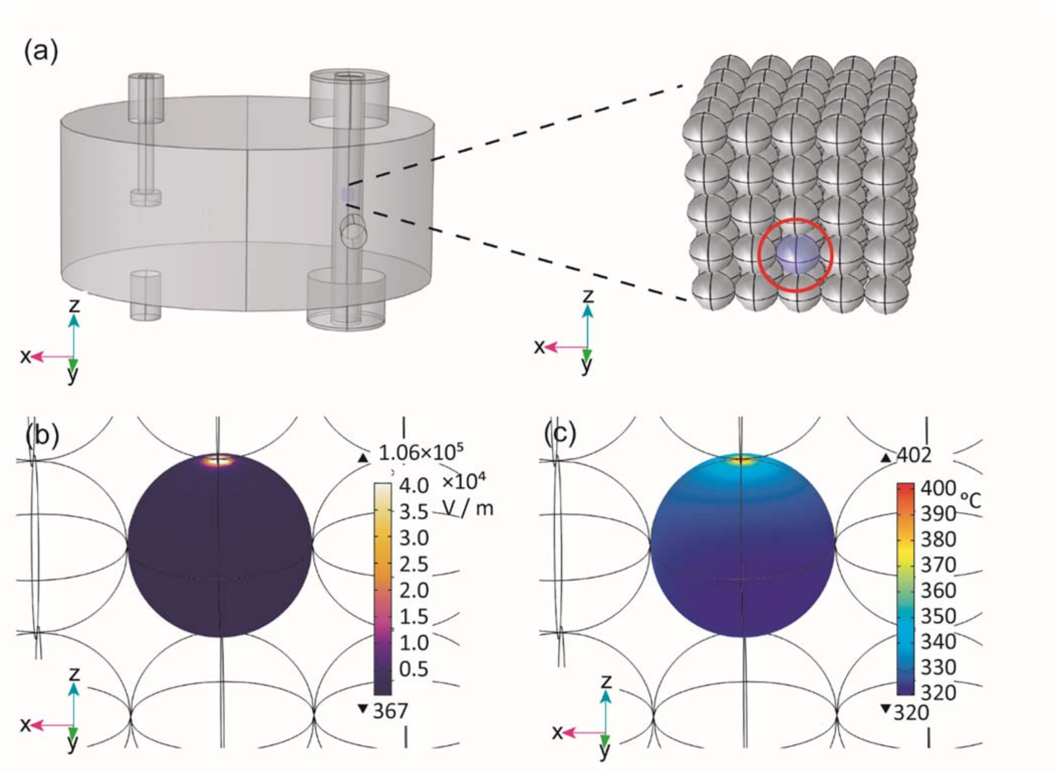

Fig. S6. (a)The simulation model duplicating the inside of the elliptical applicator including the antenna, the quartz tube, and 125 spheres with diameters of 400 μm. Simulated (b) electric field and (c) temperature distributions at the surface of one magnetite sphere indicated in a red circle in Fig. S6a. The input power was 5 W.

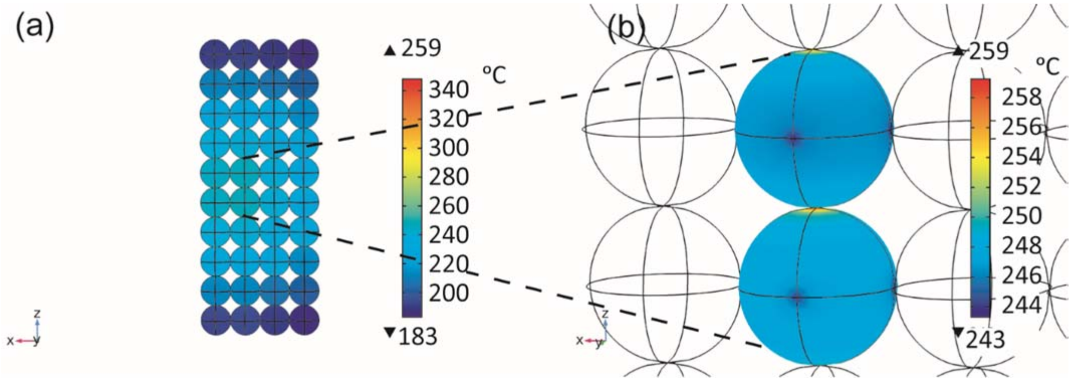

Fig. S7. Simulated temperature distributions at the surface of (a) the whole catalyst bed and (b) two magnetite catalyst spheres of C15 and C16 with a diameter of 1.9 mm in the model catalyst bed when the thermal conductivity used in the simulation was the value of the bulk. The input power was 5 W.

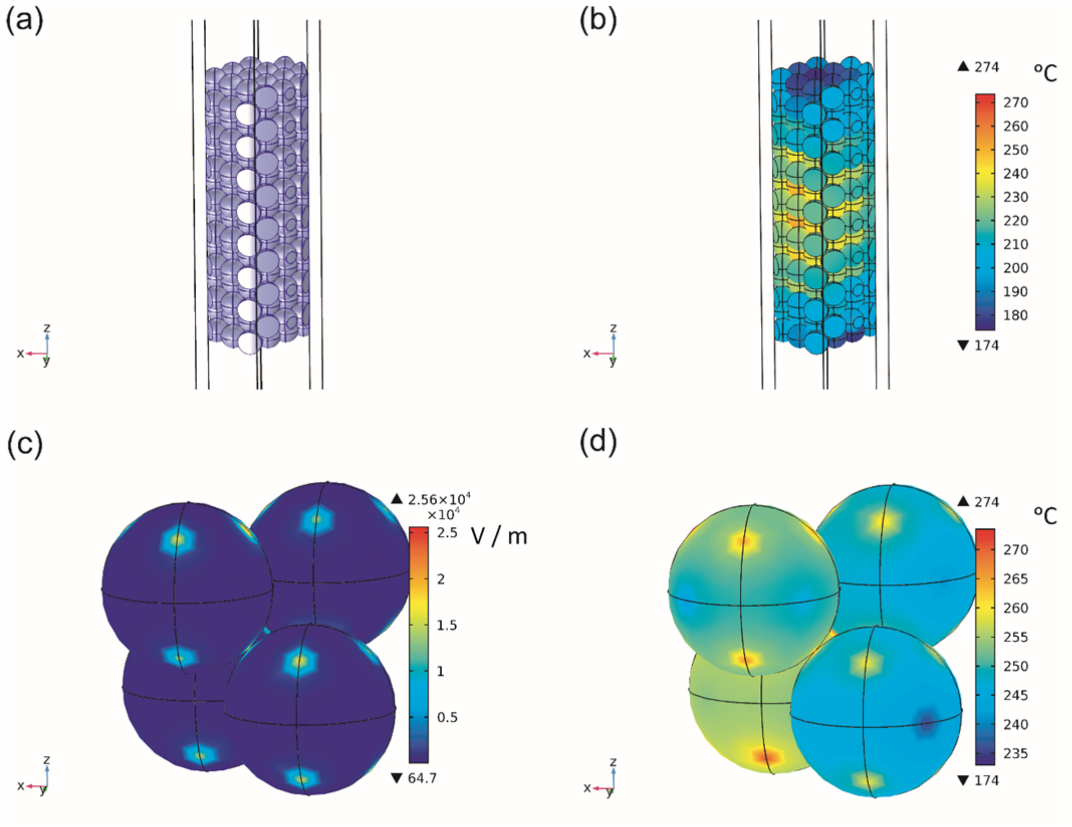

Fig. S8. (a) The simulation model of the catalyst bed, which was composed of spheres arranged in a face-centered cubic lattice. (b) Simulated temperature distributions at the surface of the catalyst bed. (c) The electric field distribution and (d) the temperature distribution at the surface of four magnetite catalyst particles with a diameter of 1.9 mm in the centre of the catalyst bed. The input power was 5 W.

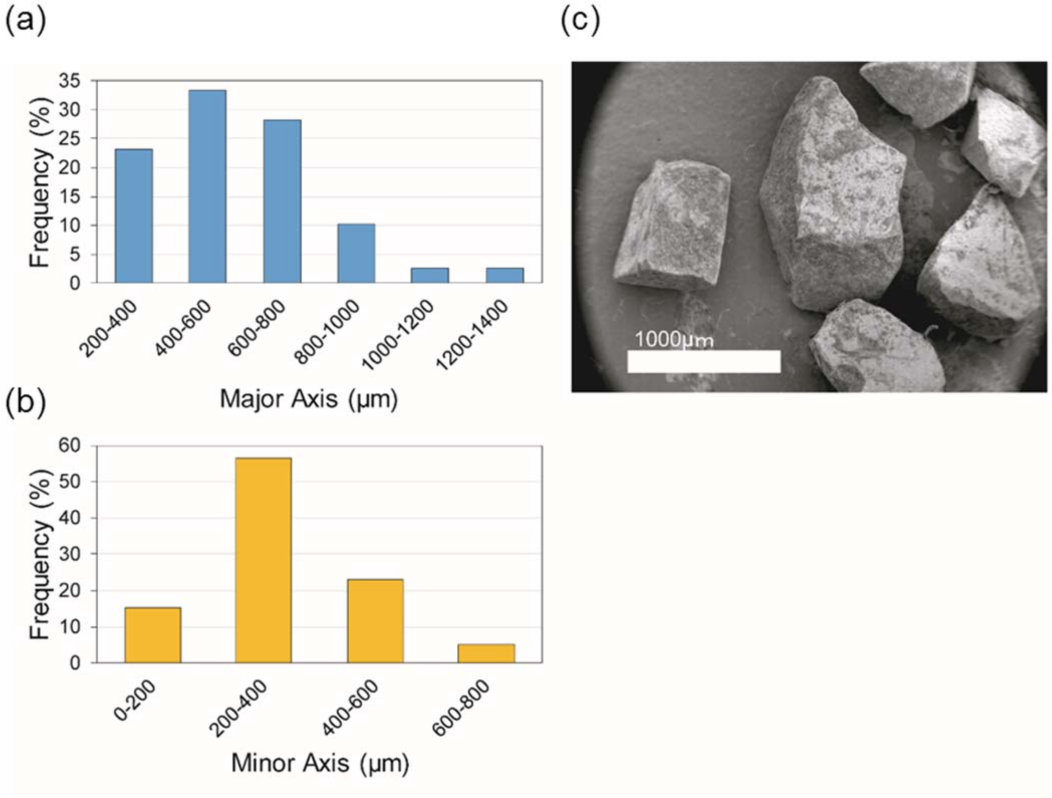

Fig. S9. The particle size distributions in (a) major axis and (b) minor axis. (c) SEM image of the magnetite catalysts.

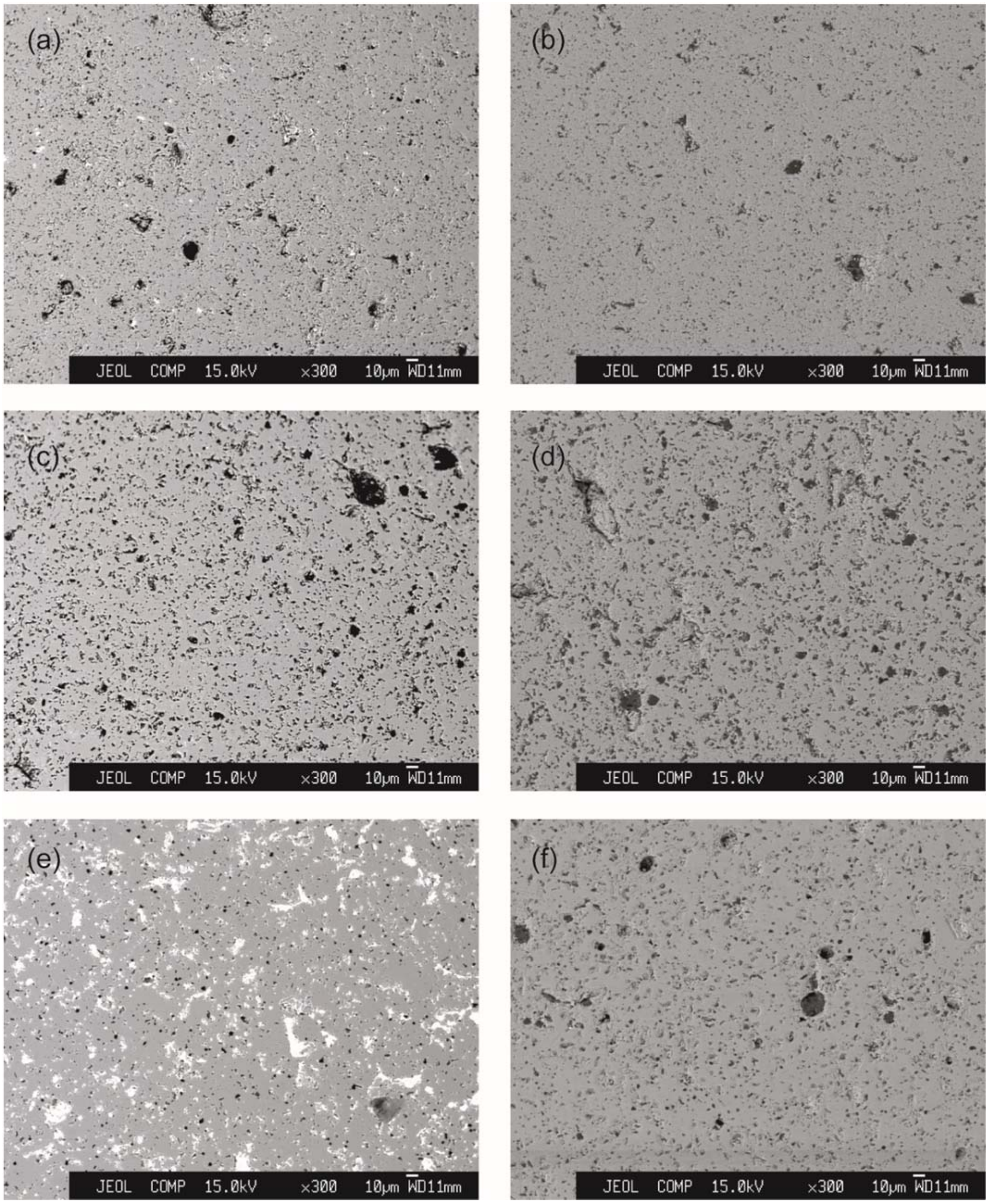

Fig. S10. EPMA images of the (a, c, e) surfaces and (b, d, f) cross sections of the spherical SiC samples. The diameters of spherical the SiC samples are (a) 2.38, (b) 3.18, and (c) 3.97 mm, respectively.
